# Trimethoprim/sulfamethoxazole‐induced methemoglobinemia in pediatric patient: A case report

**DOI:** 10.1002/ccr3.7124

**Published:** 2023-03-19

**Authors:** Sultan Alotaibi, Alaa Mously, Zainab Alnujaimy, Abdulansir Alotaibi, Abdullah Alharthi

**Affiliations:** ^1^ Pharmaceutical Care Services King Abdulaziz Medical City Jeddah Saudi Arabia; ^2^ Department of Pharmaceutical Services Prince Sultan Military Medical City Riyadh Saudi Arabia; ^3^ Department of Pediatrics Prince Sultan Military Medical City Riyadh Saudi Arabia; ^4^ Pediatric Infectious Diseases Department Women and Children's Health Institute, John Hopkins Aramco Healthcare Dhahran Saudi Arabia; ^5^ Pharmacy Services Administration King Saud Medical City, Riyadh First Cluster, MOH Riyadh Saudi Arabia

**Keywords:** high dose, methemoglobinemia, pediatrics, sulfamethoxazole/trimethoprim

## Abstract

In this report, a case of high dose of sulfamethoxazole/trimethoprim‐induced methemoglobinemia and is reported in a young boy with ventilator‐associated pneumonia and was treated successfully with methylene blue and cessation of the offending agent.

## 1 INTRODUCTION

Methemoglobinemia is a rare but potentially fatal disorder of the oxygen‐carrying capacity of hemoglobin. Here is a case representing high‐dose sulfamethoxazole/trimethoprim (SMX/TMP) induced methemoglobinemia in a young boy treated for ventilator‐associated pneumonia (VAP) due to Stenotrophomonas maltophilia, which was resolved successfully after administration of methylene blue and discontinuation of SMX/TMP.

Methemoglobinemia is considered a rare and serious disorder of the oxygen‐carrying capacity of hemoglobin and, if left untreated, can be fatal. Methemoglobinemia is defined as an increase in methemoglobin levels in the blood.^1^ The underlying mechanism includes changing the hemoglobin heme iron from a ferrous to a ferric state and eventually may lead to decrease oxygen delivery to the tissues. Methemoglobinemia can be congenital or acquired due to toxins or drugs.^2^ There are few reports of methemoglobinemia induced by traditional dosing of SMX/TMP.^3^, ^4^, ^5^, ^6^ However, in our case, methemoglobinemia occurred within 3 days from the initiation of a high dose of SMX/TMP with a rapid resolution of methemoglobinemia after discontinuation of SMX/TMP.

## 2 CASE REPORT

This is 11‐year‐old boy with a past medical history of hypothyroidism, seizure disorder and hypoxic–ischemic encephalopathy secondary to upper airway obstruction (epiglottitis) with prolonged hypoxemia, tracheostomized on mechanical ventilation at home a few years back.

The patient was admitted due to bradycardia and low potassium level. During hospitalization, the patient developed septic shock and required inotropic support for 3 days; on day 16 of admission, the patient developed pseudomonas aeruginosa ventilator‐associated pneumonia (VAP) and was treated successfully with piperacillin/tazobactam for 10 days. In between patient developed a picture of sepsis with negative cultures and received different courses of antibiotics.

On hospital day 43, he developed respiratory distress with increased oxygen requirement and doses of inotropic support. Upon further evaluation, his inflammatory markers started to elevate with remarkable chest X‐ray changes in a form of left retrocardiac airspace disease with bilateral pleural effusion. Thus, the patient was started empirically on piperacillin/tazobactam, gentamicin, and vancomycin to cover a possible hospital‐acquired infection and VAP. After 3 days, the respiratory culture showed growth of Stenotrophomonas maltophilia; therefore, the patient was started on sulfamethoxazole/trimethoprim (SMX/TMP) 5 mg/kg/every 6 h. After 3 days of initiation of sulfamethoxazole/trimethoprim, the patient had an episode of desaturation and cyanotic dusky color. The patient's oxygen saturation (SpO_2_) levels were low, with no improvement despite the ventilator setting adjustment. Conversely, the arterial blood gas (pO_2_) levels were within the normal range. The other patient's laboratory results were normal except for having metabolic acidosis with a methemoglobin level of 2.7%.

After discontinuation of SMX/TMP and administration of a methylene blue dose of 1 mg/kg intravenously for the treatment of methemoglobinemia, the methemoglobin level decreased to 0.6%, and the fraction of inspired oxygen returned to the normal range. We believe that improvement of oxygenation and methemoglobin levels after discontinuation of SMX/TMP along with administration of methylene blue is due to drug‐induced methemoglobinemia by SMX/TMP.

## 3 DISCUSSION

Methemoglobinemia is defined as an increased methemoglobin level in the blood. The underlying mechanism includes changing the hemoglobin heme iron from a ferrous to a ferric state which eventually may lead to decreased oxygen delivery to the tissues.^1^ Drugs rarely induce methemoglobinemia when given to a healthy patient. However, some of the oxidizing drugs may have the capability to convert normal hemoglobin to methemoglobin. For instance, some antimicrobials have been reported to cause methemoglobinemia, such as dapsone, sulfonamides, chloroquine, nitrofurantoin, and clofazimine.^2^ After a thorough workup and medication review, we confirmed that there were no other contributing factors for the development of methemoglobinemia except exposure to SMX/TMP.

SMX/TMP is a sulfonamide derivative that is used as an antibacterial drug for the treatment of various infectious diseases. It can be given in oral or intravenous dosage forms. SMX/TMP is rapidly and completely absorbed after oral administration with a large volume of distribution through bodily fluid. It is metabolized mainly by the liver and excreted via renal clearance. The hematological side effects of SMX/TMP include aplastic anemia, leukopenia, hemolytic anemia, thrombocytopenia, and eosinophilia.^7^

The risk factors for methemoglobinemia with oxidizing agents include patients with medical conditions such as renal failure, anemia, sickle cell disease, sepsis, lung disease, and taking large doses of SMX/TMP for a prolonged period.^2^ Because the patient developed VAP due to Stenotrophomonas maltophilia, he was started on a high dose of SMX/TMP at a dose of 5 mg per kg given every 6 h.

Methemoglobinemia is a rare disease that is difficult to diagnose. Therefore, any patient with unexplained cyanosis and hypoxia that does not improve with supplemental oxygen with normal calculated arterial pO_2_ should be suspected of methemoglobinemia. The symptoms may vary from being asymptomatic to cyanosis, dyspnea, or metabolic acidosis and may eventually lead to cardiovascular collapse and death.^8^ Similarly, our patient developed cyanosis, metabolic acidosis, and hypoxia that did not improve with increasing oxygen support.

The cornerstone of management for patients with acquired methemoglobinemia is to stop the offending agent and to establish appropriate supportive care, such as hydration for hypotension and ventilatory support for respiratory compromise. Methylene blue is considered the drug of choice for symptomatic acute methemoglobinemia. Ascorbic acid has been reported to be beneficial in methemoglobinemia and should be used when methylene blue is contraindicated or inaccessible.^8^, ^9^

We have found only four cases of methemoglobinemia induced by SMX/TMP in pediatric patients.^3^, ^4^, ^5^, ^6^ Three of them received SMX/TMP combined with another oxidizing agent and the fourth case received SMX/TMP as prophylaxis for more than 2 weeks.^5^ In our case, methemoglobinemia occurred within a few days of starting high dose of SMX/TMP therapy and was resolved rapidly after cessation of SMX/TMP therapy.

## 4 CONCLUSION

To the best of our knowledge, this is the first report of methemoglobinemia induced by a high dose of SMX/TMP in pediatric patients occurring within 3 days which may indicate methemoglobinemia due to SMX/TMP as a dose‐related side effect. Observation and rapid management are recommended.

## AUTHOR CONTRIBUTIONS

**Sultan Naif Alotaibi:** Project administration; supervision; writing – original draft. **Alaa Mously:** Data curation; investigation. **Zainab Alnujaimy:** Visualization; writing – review and editing. **Abdulnasir Alotaibi:** Project administration; writing – review and editing. **Abdullah Alharthi:** Writing – original draft; writing – review and editing.

## FUNDING INFORMATION

None.

## CONFLICT OF INTEREST STATEMENT

The authors declared that there is no conflict of interest.

## CONSENT

Written informed consent was obtained from the patient to publish this report in accordance with the journal's patient consent policy.

## Data Availability

The data that support the findings of this study are available from the corresponding author upon reasonable request.

## References

[1] Ashurst J , Wasson M . Methemoglobinemia: a systematic review of the pathophysiology, detection, and treatment. Del Med J. 2011;83(7):203‐208. 21954509

[2] Alanazi MQ . Drugs may be induced methemoglobinemia. J Hematol Thrombo Dis. 2017;5(3):1‐5.

[3] López A , Bernardo B , López‐Herce J , Cristina AI , Carrillo A . Methaemoglobinaemia secondary to treatment with trimethoprim and sulphamethoxazole associated with inhaled nitric oxide. Acta Paediatrica. 1999;88:915‐916. 10503695 10.1080/08035259950168883

[4] Jakobson B , Nilsson A . Methemoglobinemia associated with a prilocaine‐lidocaine cream and trimetoprim‐sulphamethoxazole. A case report. Acta Anaesthesiol Scand. 1985;29(4):453‐455. 4013630 10.1111/j.1399-6576.1985.tb02232.x

[5] Carroll TG , Carroll MG . Methemoglobinemia in a pediatric oncology patient receiving sulfamethoxazole/trimethoprim prophylaxis. Am J Case Rep. 2016;17:499‐502. 27424851 10.12659/AJCR.897820 PMC4950892

[6] Radhakrishnan N , Rai R . Cotrimoxazole‐induced methemoglobinemia. Indian Pediatr. 2017;54(9):786‐787. 28984267

[7] Kielhofner MA . Trimethoprim‐sulfamethoxazole: pharmacokinetics, clinical uses, and adverse reactions. Tex Heart Inst J. 1990;17(2):86‐93. 15227389 PMC326458

[8] Skold A , Cosco DL , Klein R . Methemoglobinemia: pathogenesis, diagnosis, and management. South Med J. 2011;104(11):757‐761. 22024786 10.1097/SMJ.0b013e318232139f

[9] Rino PB , Scolnik D , Fustiñana A , Mitelpunkt A , Glatstein M . Ascorbic acid for the treatment of methemoglobinemia: the experience of a large tertiary care pediatric hospital. Am J Ther. 2014;21(4):240‐243. 24914501 10.1097/MJT.0000000000000028

